# Prevalence and Outcomes of Cataract Surgery in Adult Rural Chinese Populations of the Bai Nationality in Dali: The Yunnan Minority Eye Study

**DOI:** 10.1371/journal.pone.0060236

**Published:** 2013-04-05

**Authors:** Wei Shen, Yongming Yang, Minbin Yu, Jun Li, Tao Wei, Xun Li, Juanjuan Li, Xiaodan Su, Hua Zhong, Yuansheng Yuan

**Affiliations:** 1 The First Affiliated Hospital of Kunming Medical University, Kunming, China; 2 Yunnan Red Cross Hospital, Kunming, China; 3 State Key Laboratory of Ophthalmology, Zhongshan Ophthalmic Center, Sun Yat-sen University, Guangzhou, China; 4 Kunming Medical University, Kunming, China; Zhongshan Ophthalmic Center, China

## Abstract

**Purpose:**

To investigate the prevalence and visual acuity (VA) outcomes of cataract surgery in adults of the Bai Nationality populations in rural China.

**Methods:**

We conducted a population-based cross-sectional survey (from randomly selected block groups) of Chinese Bai Nationality aged ≥50 years in southwestern China. Presenting visual acuity (PVA), best corrected visual acuity (BCVA) were recorded and a detailed eye examination was carried out. For all aphakic and pseudophakic subjects identified, information on the date, setting, type, and complications of cataract surgery were recorded. In eyes with VA <20/63, the principal cause of visual impairment was identified.

**Results:**

Of 2133 (77.8% of 2742) subjects, 99 people (129 eyes) had undergone cataract surgery. The prevalence of cataract surgery was 4.6%. Surgical coverage among those with PVA <20/200 in both eyes because of cataract was 52.8%. Unoperated cataract was associated with older age. The main barrier to cataract surgery was lack of awareness and knowledge, cost, and fear. Among the 129 cataract-operated eyes, 22.5% had PVA of ≥20/32, 25.6% had PVA of 20/40 to 20/63, 23.3% had PVA <20/63 to 20/200, and 28.7% had PVA<20/200. With BCVA, the percentages were 42.6%, 23.3%, 10.9%, and 23.3%, respectively. Aphakia (odds ratio [OR], 8.49; P<0.001) and no education (OR, 10.18; P = 0.001) or less education (OR, 6.49; P = 0.014) were significantly associated with postoperative visual impairment defined by PVA, while aphakia (OR, 8.49; P<0.001) and female gender (OR, 4.19; P = 0.004) were significantly associated with postoperative visual impairment by BCVA. The main causes of postoperative visual impairment were refractive error, retinal disorders and glaucoma.

**Conclusions:**

Half of those with bilateral visual impairment or blindness because of cataract remain in need of cataract surgery in Bai population. Surgical uptake and visual outcomes should be further improved in the future.

## Introduction

Although cataract is avoidable, it remains the leading cause of blindness worldwide, and is responsible for 47.8% or 17.7 million blind people [1]. Cataract surgery is the only currently available and effective method of restoring vision for those with visual impairment due to cataract. Population-based studies in different regional, racial and ethnic groups have suggested significant differences in cataract prevalence, surgical uptake and the quality of cataract surgeries. People in developing countries have had limited access to cataract surgery, but even if this is available the uptake of surgery can still be low. Recent population studies have reported that barriers such as cost, fear, ageism, and lack of awareness and knowledge restrict the uptake of available services [2]–[9]. Moreover, where services are available, there are concerns that they are often of poor quality [10]. Poor outcome may be due to other ocular pathology, surgical complications and uncorrected refractive error [10].

Cataract is also the leading cause of vision loss in China. However,the cataract surgical rate (CSR, cataract operations per million population per year) in China is only 446. It was almost the lowest cataract surgical rates in Asia [11]. Cataract surgical coverage (CSC) also continues to remain low [8], [9], [12]–[19]. And the quality of surgery remains a problem that should be addressed. The effect of age-related cataract can be expected to grow as the Chinese population continues to age. It is estimated that 2.5 million people in China will be blind from cataract, accounting for half of China’s blind by 2020 [20].

In recent years several population-based cross-sectional eye care studies in China have been reported [8], [9], [12]–[19], however, none focused on the Chinese ethnic minorities, whose culture, socioeconomic status, health care services accessibility, and the quality of eye care service providers may be quite different from that of the Han population.

Yunnan has a total of 26 different recognized ethnic groups. Yunnan is among the ethnically most diverse regions not only of China but also of all of Asia. The Yunnan Minority Eye Study (YMES) was designed to determine the prevalence and impact of eye diseases among rural south-western Chinese populations of the minority nationality (including the Bai, Dai, and Yi Nationality) [21], [22].

The Bai people are one of the dominant ethnic groups of Yunnan. We have recently reported the results of a 2010 survey of prevalence and causes of blindness and visual impairment in elderly Bai ethnic group in rural China [21]. The prevalence of visual impairment and blindness was 15.22% and 3.59%, respectively; Cataract accounted for 53.0% of visual impairment and 64.5% blindness. As a part of YMES, the purpose of this study was to report the prevalence of cataract surgery, surgical coverage among those visually impaired or blind as a result of cataract, risk factors for unoperated cataract, self-reported barriers to cataract surgery, visual acuity outcomes of cataract surgery, and risk factors and causes contributing to poor outcomes in adult rural Chinese populations of the Bai nationality in Dali.

## Patients and Methods

The methodology used in the YMES has been described in detail elsewhere [21], [22]. In brief, the decision to select Dali for the survey was due to the fact that more than 80% of the population of Bai nationality in China live in Dali Prefecture and it is a socioeconomic profile that is representative of Bai nationality as a whole. The subjects were identified by cluster random sampling. Those aged 50 years and older who resided in the selected study clusters for more than 6 months were considered eligible. The eligible subjects were invited for a comprehensive eye examination in the local community facilities. Written informed consent was obtained from the subjects at the examination site. A structured questionnaire was used to obtain information based on demographic data (age, gender, locality, marital status, and education), personal ophthalmic and medical history, and lifestyle (smoking and alcohol intake). Participants’ self-reported demographic data were assessed. Level of education (no school, primary school without diploma, primary school with diploma, secondary school, higher education, and higher education or above) was categorized as follows: (1) 0 year: no school; (2) 1–6 years: primary school without diploma or with diploma; (3) >6 years: secondary school or higher education or above. Subjects were asked about marital status (married, divorced, widowed, and not married). Personal ophthalmic history was investigated by means of a checklist. The subjects were asked whether they had been diagnosed with ophthalmic diseases such as cataract, glaucoma, uveitis, keratitis, ocular injury, vitreoretinopathy, and so on by a ophthalmologist. Personal medical history was also investigated by means of a checklist. The subjects were asked whether they had been diagnosed with a chronic disease such as hypertension and diabetes by a physician.

Detailed study procedures have been reported elsewhere [21], [22]. Distance VA was measured using a retroilluminated logarithm of the minimum angle of resolution tumbling E chart (wh06, Wehen Vision Technology Co., Ltd.). Each eye was measured separately, with glasses if worn for distance correction. The PVA with habitual correction was recorded. In those presenting with VA<20/25 in either eye, best-corrected visual acuity (BCVA) was assessed using the results of autorefraction and subjective refinement. For those in whom subjective refraction was not performed, BCVA was assumed to be the same as pinhole vision. The detailed examination of the eyelid, globe, pupillary reflex, lens and fundus was carried out by an experienced ophthalmologist using a slitlamp (model SL-1E; Topcon, Tokyo, Japan), a +90 diopter lens at×16 magnification and direct ophthalmoscopy.

Those with cataract surgery were queried as to the year and type of surgical facility for each operated eye. The type of cataract surgery, posterior capsule status, and signs of surgical complications were noted in the examination of cataract-operated eyes. Subjects with unoperated cataract were questioned about the main reason of not having had cataract surgery. The questionnaire presented several alternative common reasons for not having had an operation in Chinese, such as “unaware of cataract”, “could not afford to undergo cataract surgery”, “fear of further loss of vision after surgery”, “fear of surgery”, “no need to have cataract surgery”, “being told to wait until the cataract gets mature”, “not accompanied by others for surgery”, “being told that there was a contraindication”, and “undetermined”.

A principal cause of visual impairment or blindness for eyes with PVA of 20/40 or worse at presentation was determined by the examining ophthalmologist’s clinical judgment using a 15-item list (refractive error, cataract, posterior capsule opacification, phthisical/disorganized/absent globe, glaucoma, high myopic maculopathy, diabetic retinopathy, diabetic macular edema, corneal opacity/scar, age-related macular degeneration, vascular occlusion, other optic atrophy, amblyopia, other cause, undetermined cause). Refractive error was assigned as the cause for eye with of VA 20/40 or worse that could be subsequently improved to better than 20/40 with refractive correction, or with pinhole vision when subjective refraction was not possible because of age or disability. Cataract was assigned when lens opacity was commensurate with BCVA<20/25 and no other abnormality could account for the decrease in VA.

Post-operative cataract severe visual impairment or blindness was defined as cataract-operated persons who were presumed to have been bilaterally severely visually impaired or blind (PVA<20/200) when first operated on for cataract, including all bilaterally operated persons and unilaterally operated persons with a severely visually impaired or blind fellow eye. Because the indications for cataract surgery adopted by many health care facilities in rural China were cataract-induced severely visual impairment, so we presumed that the cataract-operated eyes were severe visual impairment or blindness in present study. Unoperated cataract severe visual impairment or blindness was defined as unoperated persons who were bilaterally severely visually impaired or blind (PVA<20/200) due to cataract in one or both eyes. The social burden of cataract-related visual impairment and blindness (PVA<20/200) was defined as the sum of (1) the number of persons who already had undergone surgery and who were presumed to have been bilaterally impaired or blind when first operated on for cataract, and (2) the number of unoperated persons who were bilaterally impaired or blind because of cataract in one or both eyes [15]. Cataract surgical coverage (CSC) among the cataract-related severe visual impairment or blindness (PVA<20/200) was calculated as the ratio of the post-operative presumed severe visual impairment or blindness to unoperated severe visual impairment or blindness plus the post-operative presumed severe visual impairment or blindness.

### Data Management and Analysis

The prevalence of cataract surgery in the study population was calculated by age, gender, and education. Both univariate analysis and multiple logistic regression were used to investigate the association of age, gender, and education with operated cataract, post-operative cataract with severe visual impairment or blindness, unoperated cataract with severe visual impairment or blindness, the social burden of cataract-related severe visual impairment and blindness, and CSC among the cataract-related severely visually impaired or blind population.

PVA and BCVA in cataract-operated eyes were tabulated by age, gender, and education, intraocular lens (IOL) status, and year of surgery. Visual acuity was categorized as: normal vision, ≥20/32; mild visual impairment, 20/40 to 20/63; moderate visual impairment, <20/63 to 20/200; severe visual impairment, <20/200 to 20/400; and blindness, <20/400. The association of age, gender, education, IOL status, and year of surgery with PVA and BCVA of <20/63 (considered a poor visual outcome) in post-operative eyes was investigated with univariate analysis and multiple logistic regression. Principal causes of cataract-induced visual impairment or blindness in eyes with PVA and BCVA <20/63 were tabulated.

Statistical analyses were performed using SPSS 17.0 for Windows (SPSS, Inc., Chicago, IL, USA). Confidence intervals (CIs) and P values (considered significant at the P<0.05 levels) were calculated with adjustment for clustering effects and stratification associated with the sampling design.

Ethical approval was obtained from the Kunming Medical University Ethics Review Board. The study was conducted in accordance with the tenets of the World Medical Association’s Declaration of Helsinki.

## Results

A total of 2742 subjects aged ≥50 years were enumerated, and a total of 2133 (77.8%) subjects were successfully examined. 99 persons (129 eyes) had undergone cataract surgery in one (69 subjects, 69.7%) or both eyes (30 subjects, 30.3%), representing a cataract surgery prevalence of 4.6% (95% CI, 3.8%–5.55%) (Table 1), age- and sex-standardized prevalence 3.7%. In univariate analysis, cataract surgery was significantly associated with older age and higher education. Multiple logistic regression analysis revealed that the prevalence of cataract surgery remained significantly associated with older age.

**Table 1:** Cataract Surgery by Age, Gender, and Education.

Variables	Examined	Post-operative Persons		Adjusted OR	P Value
	No. (%)	No.	% Prevalence(95% CI)	(95% CI)	
**Age(yrs)**					
50–59	715(33.5)	7	1.0 (0.3–1.7)	Reference	
60–69	775(36.3)	21	2.7 (1.6–3.9)	2.96(1.24–7.05)	0.014
70–79	525(24.6)	50	9.5 (7.0–12.0)	10.97(4.76–25.30)	0.000
80+	118(5.5)	21	17.8 (10.0–24.7)	22.36(8.93–55.99)	0.000
**Gender**					
Male	769(36.1)	35	4.6 (3.1–6.0)	Reference	
Female	1364(63.9)	64	4.7 (3.6–5.8)	1.06(0.65–1.74)	0.817
**Education(yrs)**					
>6	506(23.7)	17	3.4 (1.8–4.9)	Reference	
1–6	868(40.7)	29	3.3 (2.2–4.5)	0.68(0.36–1.28)	0.234
0	759(35.6)	53	7.0 (5.2–8.8)	0.86(0.44–1.69)	0.661
**Total**	2133(100)	99	4.6 (3.8–5.5)		

OR: odds ratios; CI: Confidence Interval.

Of the 99 post-operative subjects, 76 were presumed to have had severe visual impairment or blindness (PVA<20/200) in both eyes at the time of initial surgery (Table 2). An additional 68 subjects were severely impaired or blind because of unoperated cataract. Thus, the social burden of cataract-related severe visual impairment or blindness included 144 (6.8%) of the study participants. CSC within this cohort of post-operative and unoperated subjects was 52.8%.

**Table 2:** Post-operative and Unopreted Persons with Cataract-Related Severe Visual Impairment or Blindness (<20/200) by Age, Gender, and Education.

		Post-operative Persons				Unopreted Persons				
Variables	Examined	All Operated		Presumed Severe Visual Impairment or Blindness* ‡		Cataract Severe Visual Impairment or Blindness*		Cataract Social Burdon		Surgical Coverage (%)
	No.	No.	Prevalence†	No.	Prevalence†	No.	Prevalence†	No	Prevalence†	
**Age(yrs)**								.		
50–59	715	7	1.0	4	0.6	3	0.4	7	1.0	57.1
60–69	775	21	2.7	13	1.7	9	1.2	22	2.8	59.1
70–79	525	50	9.5	41	7.8	38	7.2	79	15.0	51.9
80+	118	21	17.8	18	15.3	18	15.3	36	30.5	50.0
**Gender**										
Male	769	35	4.6	27	3.5	25	3.3	52	6.8	51.9
Female	1364	64	4.7	49	3.6	43	3.2	92	6.7	53.3
**Education(yrs)**										
>6	506	17	3.4	10	2.0	5	1.0	15	3.0	66.7
1–6	868	29	3.3	20	2.3	22	2.5	42	4.8	47.6
0	759	53	7.0	46	6.1	41	5.4	87	11.4	52.9
**Total**	2133	99	4.6	76	3.6	68	3.2	144	6.8	52.8

* Visual acuity <20/200.

† Prevalence per 100 examined participants.

‡ Includes all bilaterally operated persons and unilaterally operated persons with a severely visually impaired or blind fellow eye.

In univariate analysis, post-operative cataract with presumed severe visual impairment or blindness, unoperated cataract with severe visual impairment or blindness and the burden of cataract-related severe visual impairment or blindness was significantly associated with older age and less education. In multiple logistic regression (Table 3), post-operative severe visual impairment or blindness, unoperated cataract severe visual impairment or blindness and the social burden of cataract-related severe visual impairment or blindness was associated with older age. However, there was no statistically significant difference between age, gender or educational status for CSC.

**Table 3:** Association of Age, Gender, and Education with Cataract-Related Severe Visual Impairment or Blindness (<20/200) by Multiple Logistic Regression Analysis.

Variables	Post-operative Presumed Severely Impaired or Blind		Unoperated Cataract Severely Impaired or Blind		Cataract Social Burdon		Cataract Surgical Coverage	
	Adjusted OR (95% CI)	P Value	Adjusted OR (95% CI)	P Value	Adjusted OR (95% CI)	P Value	Adjusted OR (95% CI)	P Value
**Age(yrs)**								
50–59	Reference		Reference		Reference		Reference	
60–69	3.01 (0.97–9.34)	0.057	2.51 (0.67–9.37)	0.171	2.80 (1.18–6.62)	0.019	1.18 (0.21–6.77)	0.855
70–79	13.19 (4.53–38.37)	0.000	14.54 (4.33–48.80)	0.000	14.75 (6.59–33.00)	0.000	0.77 (0.15–3.97)	0.753
80+	27.63 (8.86–86.16)	0.000	33.28 (9.36–118.40)	0.000	36.16 (15.25–85.73)	0.000	0.72 (0.13–4.02)	0.704
**Gender**								
Male	Reference		Reference		Reference		Reference	
Female	0.88 (0.50–1.55)	0.657	0.81 (0.44–1.46)	0.477	0.83 (0.54–1.28)	0.403	1.20 (0.52–2.76))	0.677
**Education(yrs)**								
>6	Reference		Reference		Reference		Reference	
1–6	0.81(0.37–1.78)	0.594	1.82(0.67–4.96)	0.240	1.15(0.61–2.16)	0.663	0.41(0.11–1.49)	0.177
0	1.34(0.59–3.02)	0.481	2.37(0.84–6.70)	0.104	1.76(0.91–3.38)	0.093	0.56(0.41–2.24)	0.415

OR: odds ratios; CI: Confidence Interval.

Subjects severely visually impaired or blind because of unoperated cataract cited that the most common reason for not having cataract surgery was that they were “unaware of cataract” (31 person, 45.6%), followed by “could not afford to undergo cataract surgery” (12 persons, 17.6%), “fear of further loss of vision after surgery” (5 persons, 7.4%), “fear of surgery” (4 persons, 5.9%), “no need to have cataract surgery” (4 persons, 5.9%), “being told to wait until the cataract gets mature” (4 persons, 5.9%), “not accompanied by others for surgery” (1 person, 1.5%), “being told that there was a contraindication” (1 person, 1.5%), and “undetermined” (6 person, 8.8%).

As shown, of the 129 cataract-operated eyes, 72.1% were operated on during the interval between 2001 and the time of the survey (early 2010), 20.9% were operated before 2000, and the others (7%) were uncertain. The percentage of cases that were pseudophakic was 79.8% of the 129 post-operative eyes (Table 4). The proportion of pseudophakic in cataract-operated eyes in the past 10 years was 93.5%, compared with 44.4% before 2000.

**Table 4:** Presenting and Best-Corrected Acuity of Post-operative Eyes by Age, Gender, Education, Intraocular lens Status, and Year of Surgery.

Variables		NO.(%)of Eyes	NO. (%) by Presenting Visual Acuity				NO. (%) of Best-Corrected Visual Acuity			
			≥20/32	20/40–20/63	<20/63–20/200	<20/200	≥20/32	20/40–20/63	<20/63–20/200	<20/200
**Age(yrs)**										
50–59		9 (7.0)	5 (55.6)	2 (22.2)	1 (11.1)	1 (11.1)	8 (88.9)	0 (0)	0 (0)	1 (11.1)
60–69		25 (19.4)	8 (32.0)	7 (28.0)	5 (20.0)	5 (20.0)	16 (64.0)	3 (12.0)	1 (4.0)	5 (20.0)
70–79		70 (54.3)	16 (22.9)	19 (27.1)	15 (21.4)	20 (28.6)	28 (40.0)	21 (30.0)	8 (11.4)	13 (18.6)
80+		25 (19.4)	0 (0)	5 (20.0)	9 (36.0)	11 (44.0)	3 (12.0)	6 (24.0)	5 (20.0)	11 (44.0)
**Gender**										
Male		47 (36.4)	13 (27.7)	15 (31.9)	10 (21.3)	9 (19.1)	24 (51.7)	14 (29.8)	2 (4.3)	7 (14.9)
Female		82 (63.6)	16 (19.5)	18 (22.0)	20(24.4)	28 (34.1)	31 (37.8)	16 (19.5)	12 (14.6)	23 (28.0)
**Education**										
0		68 (52.7)	11 (16.2)	14 (20.6)	18 (26.5)	25 (36.8)	21 (30.9)	17 (25.0)	11 (16.2)	19 (27.9)
1–6		39 (30.2)	6 (15.4)	13 (33.3)	11 (28.2)	9 (23.1)	17 (43.6)	10 (25.6)	3 (7.7)	9 (23.1)
>6		22 (17.1)	12 (54.5)	6 (27.3)	1 (4.5)	3 (13.6)	17 (77.3)	3 (13.6)	0 (0)	2 (9.1)
**IOL status**										
Aphakic		26 (20.2)	1 (3.8)	1 (3.8)	5 (19.2)	19 (73.1)	1 (3.8)	7 (26.9)	4 (15.4)	14 (53.8)
Pseudophakic		103 (79.8)	28 (27.2)	32 (31.1)	25 (24.3)	18 (17.5)	54 (52.4)	23 (22.3)	10 (9.7)	16 (15.5)
**Year of surgery**										
≥2001		93 (72.1)	26 (28.0)	28 (30.1)	22 (23.7)	17 (18.3)	48 (51.6)	22 (23.7)	9 (9.7)	14 (15.1)
≤2000		27 (20.9)	3 (11.1)	5 (18.5)	6 (22.2)	13(48.1)	6 (22.2)	8 (29.6)	4 (14.8)	9 (33.3)
Missing		9 (7.0)	0 (0)	0 (0)	2 (22.2)	7 (77.8)	1 (11.1)	0 (0)	1 (11.1)	7 (77.8)
**Total**	129 (100)		29 (22.5)	33 (25.6)	30 (23.3)	37 (28.7)	55 (42.6)	30 (23.3)	14 (10.9)	30 (23.3)

IOL: intraocular lens.

Of the 99 post-operative subjects, 30 were operated in both eyes: 21 were pseudophakic in both eyes, 5 were aphakic in both eyes, and 4 were pseudophakic in one eye and aphakic in the fellow eye. For the 69 with unilateral cataract surgery, 57 were pseudophakic and 12 were aphakic. Five (5.1%) of the 99 post-operative subjects were wearing glasses for distance correction at the time of the examination.

PVA and BCVA for the 129 post-operative eyes by age, gender, education, IOL status, and year of surgery are shown in Table 4. Overall, a reasonably good vision outcome (VA ≥20/63) was found in 48.1% of eyes with PVA and in 65.9% with BCVA. In univariate analysis, higher prevalence of visual impairment (VA <20/63) defined by both PVA and BCVA was significantly associated with older age, female, less education, aphakia, and year of surgery. In multiple logistic regression (Table 5), aphakia (odds ratio [OR], 8.49; P<0.001) and no education (OR, 10.18; P = 0.001) or less education (OR, 6.49; P = 0.014) were significantly associated with postoperative visual impairment by PVA, while aphakia (OR, 8.49; P<0.001) and female gender (OR, 4.19; P = 0.004) were significantly associated with postoperative visual impairment by BCVA. Age or year of surgery was not significantly associated with either PVA or BCVA, in part because of its correlation with IOL status.

**Table 5:** Effect of Variables on Presenting and Best-Corrected Visual Acuity <20/63 in Post-operative Eyes by Multiple Logistic Regression Analysis.

Variables	Presenting Visual Acuity				Best-Corrected Visual Acuity			
	P Value	Regression Coefficient	OR	95% CI	P Value	Regression Coefficient	OR	95% CI
**Age(yrs)**								
50–59	Reference				Reference			
60–69	0.910	0.114	1.12	0.16–8.03	0.742	0.407	1.50	0.13–17.03
70–79	0.964	−0.046	0.96	0.13–6.92	0.980	0.031	1.03	0.09–12.51
80+	0.298	1.147	3.15	0.36–27.36	0.329	1.267	3.55	0.28–45.29
**Gender**								
Male	Reference				Reference			
Female	0.807	0.131	1.14	0.40–3.26	0.004	1.432	4.19	1.59–11.04
**Education**								
>6	Reference				Reference			
1–6	0.014	1.870	6.49	1.46–28.89	0.494	0.658	1.93	0.29–12.73
0	0.001	2.320	10.18	2.43–42.61	0.204	1.263	3.54	0.50–24.81
**IOL status**								
Pseudophakia	Reference				Reference			
Aphakia	<0.001	3.028	20.66	4.06–105.14	<0.001	2.139	8.49	3.01–23.95
**Year of surgery**								
≤2000	Reference				Reference			
≥2001	0.577	−0.352	0.70	0.20–2.42	0.547	−0.374	0.69	0.20–2.33

OR: odds ratios; CI: Confidence Interval; IOL: intraocular lens.

Table 6 identifies the principal cause of impairment for the 67 cataract-operated eyes with VA worse than 20/63. The main causes of post-operative visual impairment were refractive error, retinal disorders and glaucoma. Potentially treatable diseases, including refractive error, PCO, and pterygium accounted for 46.3% of impaired cataract-operated eyes.

**Table 6:** Principal Cause of Presenting and Best-Corrected Visual Acuity <20/63 in Post-operative Eyes.

Principal Cause	Presenting Visual Acuity			Best-Corrected Visual Acuity
	Aphakia	Pseudophakia	Total	
Refractive error/uncorrected aphakia	5(20.8)	17(39.5)	22 (32.8)	0 (0.0)
Retinal disorder	7(29.1)	5(11.6)	12 (17.9)	12(27.3)
AMD	4(16.7)	4(9.3)	8 (11.9)	8 (18.2)
Retinal detachment	1(4.2)	0(0.0)	1 (1.5)	1 (2.3)
Macular hole	0(0.0)	1(2.3)	1 (1.5)	1 (2.3)
Vitreous haze	2(8.3)	0(0.0)	2 (3.0)	2 (4.5)
Glaucoma	2(8.3)	8(18.6)	10 (14.9)	10 (22.7)
Optic atrophy	6(25.0)	2(4.7)	8 (11.9)	8 (18.2)
PCO	1(4.2)	4(9.3)	5 (7.5)	5 (11.4)
pterygium	2(8.3)	2(4.7)	4(6.0)	4 (9.0)
High myopic retinopathy	1(4.2)	2(4.7)	3(4.5)	2 (4.5)
Corneal opacity/scar	0(0.0)	1(2.3)	1 (1.5)	1 (2.3)
Other cause	0(0.0)	1(2.3)	1 (1.5)	1 (2.3)
Undetermined	0(0.0)	1(2.3)	1 (1.5)	1 (2.3)
All	24(100)	43(100)	67 (100)	44 (100)

AMD: age-related macular degeneration; PCO: posterior capsule opacification.

Data are presented as No. (%) of eyes.

## Discussion

This study provides the first population-based data on the prevalence of cataract surgery, surgical coverage, risk factors for unoperated cataract, self-reported barriers to cataract surgery, visual acuity outcomes of cataract surgery, and risk factors and causes contributing to poor outcomes in the elderly Bai ethnic population in rural China. Although the sample size chosen for the survey was calculated according to estimates of prevalence of glaucoma, we have no reason to suspect that the sample of operated cases was not representative of the Bai as a whole.

The prevalence of cataract surgery of 4.6% as found among the Bai people in current study was considerably higher than that found earlier in China [8], [9], [12]–[15], in Sri Lanka (3.9%) [3], and lower than Brazil (6.28%) [23], Nepal (7.0%) [24], India (17.6%) [25]. These figures suggest a pronounced increase in the prevalence of cataract surgery in the past 15 years in rural China. Since minorities reside in the remote area and usually poor, the Chinese government has a preference policy to provide funding and training for the minorities to progress, govern and manage. The encouraging prevalence of cataract surgery may be attributed to rapid economic growth in Dali, and sustained successful effort on sight-restoring cataract surgery by training cataract surgeons, inputting surgical equipment, and offering free surgeries in Yunnan in recent years.

CSC of 52.8% as found among the Bai people in current study was generally comparable to that reported in Shunyi (47.8%) [12], Kunming (46.4%) [9], Tibet (56%) [8], and higher than that reported in Doumen (40.3%) [13] and the China Nine-Province Survey (35.7%) [15], but lower than Beijing (82.1%) [14]. CSC found in Dali was also at the moderate level, compared with previous studies in other developing countries [3]–[7], [23]–[27]. The moderate CSC in Bai people reveals that combating cataract blindness just now gained a preliminary victory and that sustained efforts are needed to reach the remaining half of Bai people with bilateral visual impairment or blindness because of cataract. Findings also suggest that the CSC showed a more equitable distribution of cataract surgical service delivery by gender and educational status. A similar trend was observed for CSC in Harbin [16].

The social burden of cataract-related severe visual impairment or blindness of 6.8% as found among the Bai people in current study was remarkably higher than that found among the Han people in Beijing (3.48%) [14] and the China Nine-Province Survey (3.83%) [15]. A greater social burden of cataract-related severe visual impairment or blindness is suggestive a great incidence of cataract-related severe visual impairment or blindness, which may be due to a greater risk of cataract development. The elderly were particularly affected by severe visual impairment or blindness because of both unoperated and post-operative cataract. However, CSC differentially favored younger people, although not at level of statistical significance. The rapidly ageing population of Bais and the higher altitude (1974 m) with increased exposure to ultraviolet light in Dali may account for the higher burden of visual impairment or blindness resulting from cataract that was seen in our study. Education of the elderly within the community may therefore bring about a more considerable reduction in the burden of visual impairment or blindness in the region.

Current findings suggest the most commonly self-reported barriers to surgery in Bai population were lack of awareness and knowledge, cost and fear. Public health education to raise public awareness of the importance of eye health will need to take account of the very high levels of illiteracy found among the Bai people with unoperated cataract visual impairment or blindness (60.5%). By improving health education on cataract, reducing the price of cataract surgery for those who cannot afford it, and ensuring a high surgical quality, the uptake of cataract surgery could be increased considerably.

According to World Health Organization guidelines [28], visual outcome of cataract surgery among Bai people in our study was generally poor. The proportion of eyes with PVA and BCVA of 20/63 or better was 48.1% and 65.9%, respectively. The visual outcome in our study is comparable that found in the China Nine-Province Survey [15] and India [25], is better than that in earlier China [9], [12], [13], [16] and Pakistan [29], and is worse than that in economically developed areas [14], [17], [18]. This result is partly due to differences in the study population and quality of eye care service.

The risk factors associated with visual impairment in post-operative adult Bai people include aphakia, illiteracie and female. The strongest association of poor visual acuity outcomes was aphakia; in multiple logistic regression, aphakia was 20 times more likely than pseudophakia to have poor outcome. Findings suggest that aphakia was more likely to suffer from surgical complications. Although no other abnormality could account for the decrease in VA, uncorrected aphakia were not fully correctable with corrective spectacles due to underlying surgical complications or optical weaknesses present in spectacles. We also found the presence of optic atrophy in 7 subjects (8 eyes) who had poor outcome after cataract surgery. Of the 7 such cases, 1 was bilateral aphakia in eyes with cataract surgery, while 6 was unilateral in eyes with cataract surgery, 4 aphakia, and 2 pseudophakic. Dandona et al. [30] suspect that the unilateral optic atrophy after cataract surgery with no other obvious cause may result from anesthesia or poor surgical technique. Fortunately, the proportion of IOL implantation in Dali was much higher than some that have been reported in similar surveys in China [8], [9], [12], [13], [15], [16], and the value in the past 10 years was markedly higher than that before 2000. This may reflect the improvement of surgical technique in the region. The relatively poor PVA outcomes in illiteracies were the result of more cases with refractive error, retinal disorders, glaucoma, and other ocular comorbidities. And the relatively poor BCVA outcomes in females were the result of more cases with retinal disorders, glaucoma, and PCO. Illiteracy and female gender were used as disadvantage groups for socioeconomic status and members of these groups may mistakenly attribute poor visual acuity to failure of cataract surgery and may not seek future treatment for refractive error, PCO, or other ocular disease.

The main causes of visual impairment in post-operative eyes of the Bai people were refractive error, retinal disorders, and glaucoma. Similar findings were reported from surveys from Doumen [13], Liwan [17], and Hong Kong [18]. Potentially treatable diseases, including refractive error, PCO, and pterygium accounted for 46.3% of impaired eyes. Refractive error was the most common cause. The use of spectacles was only 5.1% among cataract-operated subjects. Pseudophakia accounted for more than three fourths of those with refractive error. The differences in PVA and BCVA after operation highlight the importance of appropriate preoperative IOL power estimation and postoperative adequate spectacle provision. PCO is a well-recognized, vision-impairing complication of cataract surgery and can be treated easily with laser capsulotomy. Pterygium, the prevalence of which was high in the Bais, can also be diagnosed and resolved easily with out-patient surgery. We could not determine whether retinal disorders and glaucoma had coexisted at the time of cataract surgery or developed after cataract surgery. Some pathologic features may be surgery-related, such as retinal detachment, partial AMD, which presented in aphakic eyes. To avoid these problems, more attention should be given to thorough preoperative screening, high cataract surgical quality, regular postoperative follow-up, and timely treatment of complications and comorbidities.

There still remain some limitations in our study. Firstly, the relatively low response rate of the youngest group (aged 50–59 years, 63.7%) and male (58.5%) [22] was a source of potential bias. Younger men who were more healthy and mobile may have been less likely to participate because they were busy with working. Under-representation in younger men might result in overestimation of the true burden of cataract-related severe visual impairment and blindness. Secondly, similar to previous studies in China, cataract was diagnosed not using a standardized cataract classification system. We adopted the clinical diagnostic criteria of cataract for which does not affect the analysis of cataract surgical coverage among the cataract severely impaired or blind population.

In conclusion, cataract surgery is reaching more than half of those with bilateral severe visual impairment or blindness because of cataract in the elderly Bai population living in rural south-western China. Surgical uptake is still low, and the social burden of cataract-related severe visual impairment or blindness is heavy, especially in the elderly. A significant degree of poor outcomes remains after cataract surgery. Many of the potential causes of visual impairment are treatable to improvement or correction by careful postoperative follow-up. Surgical uptake and visual outcomes should be further improved by creating a program that facilitates access to ophthalmic healthcare services by Bai people would address many of the risk factors and causes associated with visual impairment in cataract-operated adult Bai people.
